# 

**Published:** 1929

**Authors:** 


### Contents of Vol. XLVI.

*Page*

Some Points on the Care and Treatment of Mental Defectives. By H. L. Ormerod, M.D., B.Ch., M.R.C.S., L.R.C.P. .. .. .. .. 1

Radium and its Surgical Applications. By H. S. Souttar, C.B.E., F.R.C.S. .. .. .. .. .. .. .. .. .. 19

The Distal Phenomena that accompany High Arterial Tension. By Carey F. Coombs, M.D., F.R.C.P. .. .. .. .. .. .. 35

The Rôle of Pyelography in Renal Disorders. By A. Wilfrid Adams, M.S., F.R.C.S. .. .. .. .. .. .. .. .. 49

The Management of the Normal Puerperium. By H. J. Drew Smythe, M.M., M.B., M.S., F.R.C.S. .. .. .. .. .. .. 59

A Case of Rectal Prolapse. By D. Robertson, M.B., Ch.B., and D. G. C. Tasker, M.S., F.R.C.S. .. .. .. .. .. .. 71

Foreign Bodies in the Gullet. By E. Watson-Williams, M.C., Ch.M., F.R.C.S. .. .. .. .. .. .. .. .. .. 109

A Case of Primary Sarcoma of the Spleen. By Arthur L. Taylor, M.D. 121

Clinical Diagnosis of Primary Syphilis. By A. Michael Critchley, M.D. 127

Portal Pyæmia following Diverticulitis, with Report of a Case. By Frank Bodman, M.B., M.R.C.S., and Arthur L. Taylor, M.D. .. .. 131

An Epidemic of Virulent Diphtheria. By Thomas Aubrey, M.B., and R. B. Britton, M.B., B.S. .. .. .. .. .. .. 141

The Histology of the Aortic Wall in Acute Rheumatism. By J. J. Giraldi 145

The Significance of Sleep Disorders. By Hugh H. Carleton, M.D., M.R.C.P. .. .. .. .. .. .. .. .. .. 173

The Borderland between Surgery and Gynæcology. By E. W. Hey Groves, M.S., F.R.C.S. .. .. .. .. .. .. .. .. 185

Anæsthetics. By Stuart V. Stock, M.B., B.S., M.B., B.Ch., F.R.C.S. 197

The Association between Angioma of the Cerebellum, Polycystic Pancreas and Renal Adenoma (Lindau's Syndrome). By Geoffrey Hadfield, M.D., M.R.C.P. .. .. .. .. .. .. .. .. 211

Senile Arterial Changes in a Child Aged Seven Weeks. By F. W. Terrell Hughes, M.B., Ch.B., and C. Bruce Perry, M.D., M.R.C.P. .. .. 219

The Long Fox Memorial Lecture: Ten Years' Progress in Surgical Treatment. By A. Rendle Short, M.D., F.R.C.S. .. .. .. 243

The Treatment of Parenchymatous Goitre. By W. A. Jackman, M.B., Ch.B., F.R.C.S.E. .. .. .. .. .. .. .. .. 277

Three Cases of Sino-Auricular Block. By H. L. Heimann, M.D., M.R.C.P. 285

The Early Treatment and Diagnosis of Anterior Poliomyelitis. By R. G. Gordon, M.D., D.Sc., F.R.C.P.E... .. .. .. .. .. 289

Reviews of Books .. .. .. .. .. .. 77, 163, 223, 299

Editorial Notes .. .. .. .. .. .. .. 90, 167, 231, 310

Obituaries .. .. .. .. .. .. .. .. .. 168, 238

Meetings of Societies .. .. .. .. .. .. .. 91, 169, 313

The Medical Library of the University of Bristol .. 97, 170, 240, 335

Local Medical Notes .. .. .. .. .. .. 99, 171, 241, 337

